# Repeatability of corneal elevation maps in keratoconus patients using the tomography matching method

**DOI:** 10.1038/s41598-017-17658-7

**Published:** 2017-12-12

**Authors:** YaRu Zheng, LiFang Huang, YiPing Zhao, JunJie Wang, XiaoBo Zheng, Wei Huang, Brendan Geraghty, QinMei Wang, ShiHao Chen, FangJun Bao, Ahmed Elsheikh

**Affiliations:** 1 Eye Hospital, Wenzhou Medical University, Wenzhou, 325027 China; 2 QuanZhou Women’s and Children’s Hospital, QuanZhou, 362000 China; 3 The institution of ocular biomechanics, Wenzhou Medical University, Wenzhou, 325027 China; 4 School of Engineering, University of Liverpool, Liverpool, L69 3GH UK; 5 The National Institute for Health Research (NIHR) Biomedical Research Centre for Ophthalmology, Moorfields Eye Hospital NHS Foundation Trust and UCL Institute of Ophthalmology, London, UK

## Abstract

To assess repeatability of corneal tomography in successive measurements by Pentacam in keratoconus (KC) and normal eyes based on the Iterative Closest Point (ICP) algorithm. The study involved 143 keratoconic and 143 matched normal eyes. ICP algorithm was used to estimate six single and combined misalignment (CM) parameters, the root mean square (RMS) of the difference in elevation data pre (PreICP-RMS) and post (PosICP-RMS) tomography matching. Corneal keratometry, expressed in the form of M, J_0_ and J_45_ (power vector analysis parameters), was used to evaluate the effect of misalignment on corneal curvature measurements. The PreICP-RMS and PosICP-RMS were statistically higher (P < 0.01) in KC than normal eyes. CM increased significantly (p = 0.00), more in KC (16.76 ± 20.88 μm) than in normal eyes (5.43 ± 4.08 μm). PreICP-RMS, PosICP-RMS and CM were correlated with keratoconus grade (p < 0.05). Corneal astigmatism J_0_ was different (p = 0.01) for the second tomography measurements with misalignment consideration (−1.11 ± 2.35 D) or not (−1.18 ± 2.35 D), while M and J_45_ kept similar. KC corneas consistently show higher misalignments between successive tomography measurements and lower repeatability compared with healthy eyes. The influence of misalignment is evidently clearer in the estimation of astigmatism than spherical curvature. These higher errors appear correlated with KC progression.

## Introduction

Keratoconus (KC), is a non-inflammatory progressive condition of the cornea and the most prevalent form of idiopathic corneal ectasia. It is characterized by localized thinning and conical protrusion of the cornea which results in regular and irregular astigmatism and decrease in visual quality^1^. Thinning of the cornea is initially found in the inferior-temporal and central zones^2^ although superior localizations can also occur^3^. The progression and severity of keratoconus can be monitored by measuring the distribution of corneal thickness and the degree of protrusion.

Periodic corneal shape monitoring is currently the main method adopted to determine the progression of corneal thinning and protrusion in KC, and the effectiveness of management techniques such as collagen cross-linking (CXL) and rigid gas permeable lens wear in halting progression. Various corneal shape measurement methods exist including the Placido^4,5^, Scheimpflug^6^^,^^7^, and Optical coherence tomography (OCT)^8,9^, all of which need to comply with strict repeatability criteria in order to provide reliable information on progression. Here the typically irregular surface of the keratoconic cornea presents a difficult challenge to achieving good repeatability of tomography measurements. A possible complication is that most tomography methods provide elevation data at a set of regularly-spaced discrete points, and therefore misalignment between successive measurements (either taken in the same setting to check repeatability or separated by a time period to check progression) can mean a different set of points is measured every time, leading to considerable differences in results. This study attempts to assess the effectiveness of a surface matching technology – an Iterative Closest Point (ICP) algorithm, developed in an earlier study^10,11^. As a feature-based surface matching technique and the dominant method for image registration, ICP checks the similarities between overlapping maps to determine the rigid-body transformations needed for the best possible match. ICP was employed in this study to estimate and correct for misalignment between successive tomography measurements in KC and normal humans, and determine the effect of misalignment, before and after correction, on repeatability of tomography data.

## Results

There was a wide range of BCVA (0.0 to 1.4, and −0.2 to 0.1) for KC and normal eyes, respectively. BCVA in KC was worse than in normal group (p < 0.01). The mean values of RE were −5.10 ± 4.32 D (−19.50 ~ +4.50 D), −4.49 ± 2.03 D (−10.50 ~ +0.50 D) for the spherical component, and −4.12 ± 2.23 D (−8.75 ~ 0.00 D), −0.81 ± 0.55 D (−2.75 ~ 0.00 D) for the cylindrical component in KC and normal eyes, respectively.

**Figure 1:**
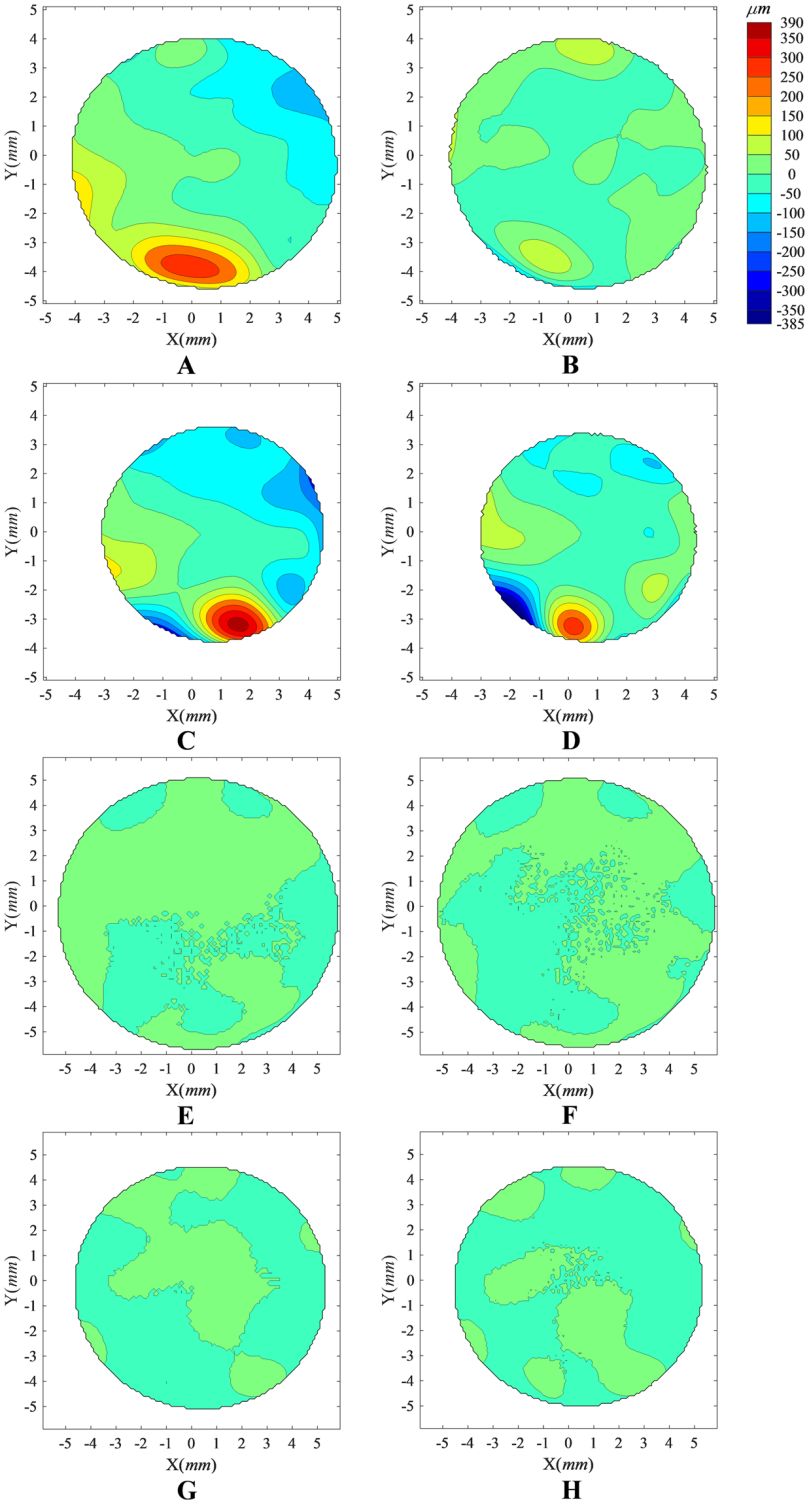
Distribution of elevation differences between successive corneal topography maps recorded before and after elimination of misalignment using ICP algorithm. The analysis was carried out for a randomly-selected KC case (**A**–**D**) and a gender- and age-matched (age difference less than 5 years) Normal case (**E**–**H**). Contour maps (**A**,**B**,**E**,**F**) show the elevation differences in the common region of two successive anterior corneal topographies recorded before (**A**,**E**) and after (**B**,**F**) elimination of misalignment, while contour maps (**C**,**D**,**G**,**H**) show corresponding elevation differences in the common region of posterior topographies recorded before and after elimination of misalignment. The eight contour maps share the same colour scale (upright in μm). Before ICP correction of misalignment in the KC case, the RMS of fit error was 87.11 μm for both anterior and posterior surfaces, considered simultaneously, and reduced to 52.39 μm following the ICP correction. This can be compared to the Normal case where the RMS of fit error before ICP correction was 9.09 μm for both anterior and posterior surfaces, considered simultaneously, and reduced to 6.64 μm following the ICP correction.

Tomography matching results are shown in Tables 1 and 2. Representative images of KC case and normal case were provided in Fig. 1. After correcting for misalignment, PosICP-RMS was significantly lower than PreICP-RMS in both anterior and posterior surfaces and in both KC and normal eyes (p < 0.01). The PreICP-RMS, PosICP-RMS and the misalignment ratio were significantly higher in the KC group compared with the control group (p < 0.01, Table 2). All of the misalignment parameters (x_0_, y_0_, z_0_, α, β, γ) between successive measurements were not significantly different in the KC group compared to the control group (p > 0.05, Table 3), although CM was significantly higher in the KC group than in the control group (p < 0.01).

**Table 1:** Matching errors between successive tomography measurements for keratoconic and normal eyes.

Group	Corneal surface	PreICP-RMS, μm	PosICP-RMS, μm	Misalignment ratio, %
Control	Anterior	5.12 ± 3.07	2.83 ± 1.12	38.92 ± 17.59
	Posterior	12.66 ± 5.20	11.08 ± 4.72	12.54 ± 11.40
	Anterior	18.43 ± 21.54	6.35 ± 4.58	55.20 ± 19.99
Keratoconus	Posterior	29.53 ± 24.62	19.62 ± 11.79	27.01 ± 16.83

PreICP-RMS and PosICP-RMS represent the root-mean-square of the elevation data obtained for corneal surfaces in successive measurements and presented both before and after tomography matching; Misalignment ratio = 1 - (PosICP-RMS/PreICP-RMS).

**Table 2:** Comparison of matching error results of the first and second measurement between keratoconus and control groups.

Corneal surface	PreICP-RMS, μm	PosICP-RMS, μm	Misalignment ratio
Anterior	0.000**	0.000**	0.000**
Posterior	0.000**	0.000**	0.000**

Mann-Whitney U test was used to compared the tomography matching results of control and keratoconus groups. PreICP-RMS and PosICP-RMS represent the root-mean-square error of the coordinate differences of corneal surface between two successive measurement before and after tomography matching, respectively; Misalignment ratio = 1- (PosICP-RMS/PreICP-RMS); *means P < 0.05, ** means P < 0.01.

**Table 3:** Translational and rotational misalignments between successive tomography measurements.

Group	α, degree	β, degree	γ, degree	x_0_, μm	y_0_, μm	z_0_, μm	CM, μm
Control	−0.04 ± 0.77	0.09 ± 0.45	−0.37 ± 2.42	12.49 ± 60.77	4.36 ± 99.77	−0.85 ± 3.29	5.43 ± 4.08
Keratoconus	−0.07 ± 0.88	0.14 ± 0.75	−0.21 ± 3.81	16.1 ± 81.4	5.14 ± 85.34	−1.42 ± 4.7	16.76 ± 20.88
Comparison	0.527	0.518	0.053	0.662	0.699	0.171	0.000**

Mann-Whitney U test was used to compared the tomography matching results of control and keratoconus groups; α, β, γ represent the rotational misalignments about the three main axes x, y and z, respectively, calculated for both the anterior and posterior corneal surfaces; x_0_, y_0_, z_0_ represent the translational displacements of anterior and posterior corneal surfaces; Combined misalignment parameter (CM) was developed to combine the effects of all six misalignment components; * means P < 0.05, ** means P < 0.01.

The median of keratoconus grade was 3 with a range 1 to 4. Further, in both corneal surfaces of KC eyes, PreICP-RMS and PosICP-RMS were correlated with KC grade, and the correlations were much stronger in the anterior surface (r = 0.57, 0.55, respectively) than in the posterior surface (r = 0.51, 0.41). For the misalignment ratio, while it remained correlated with KC grade, the correlation was stronger in the posterior surface (r = 0.26) than in the anterior surface (r = 0.21) (Table 4). Further, CM was also significantly correlated with the KC grade (r = 0.48) even though the individual misalignment parameters (x_0_, y_0_, α, β, γ) did not show significant correlation (r = 0.06, 0.03, −0.06, 0.15, −0.07, respectively) with KC grade except for z_0_ (r = −0.20).

**Table 4:** Correlation of keratoconus grade with matching error results of two successive tomography measurements.

Periods	Corneal surface	PreICP-RMS (μm)	PosICP-RMS (μm)	Misalignment ratio (%)
Keratoconus grade	Anterior	0.57**	0.55**	0.21**
	Posterior	0.51**	0.41**	0.26**

PreICP-RMS and PosICP-RMS represent the root-mean-square differences between the elevation data of two successive measurements taken before and after tomography matching; Misalignment ratio = 1 - (PosICP-RMS/PreICP-RMS); Keratoconus grade is based on the Tomographic Keratoconus Classification system (TKC) provided by the Pentacam software, which allows classification into 5 grades: 0 (normal) to 4 (severe keratoconus). * means P < 0.05, **means P < 0.01.

Further, while M, J_0_ and J_45_, obtained before tomography matching were 51.10 ± 6.21 D, −1.18 ± 2.35 D and −0.13 ± 1.50 D, respectively, they slightly changed to 51.08 ± 6.20 D, −1.11 ± 2.35 D and −0.11 ± 1.56 D after correction. These changes were significant in only the case of J_0_ (p = 0.01) but were insignificant in M (p = 0.64) and J_45_ (p = 0.53).

## Discussion

Corneal shape assessment has evolved over the last few decades and is used extensively now in the diagnosis, staging and follow-up of keratoconus^12^ and planning of refractive surgeries^13^. It provides anterior, and in some instruments posterior, surface tomography of the cornea that is derived from true elevation measurements^14^. The accuracy and repeatability of tomography measurements assume growing importance with the advent of new prophylactic and therapeutic corneal interventions such as intrastromal corneal ring segment implantation^15^, collagen crosslinking^16^, and deep lamellar keratoplasty^17^. The planning of these applications relies on elevation data that is reliable and repeatable within a few microns. This requirement is addressed in our study where the repeatability of corneal elevation measurements is assessed in both keratoconus patients and healthy controls using the Pentacam, which based on the Scheimpflug technology.

The literature showed the Scheimpflug system to have excellent repeatability in measuring corneal curvature in normal eyes^18^ but uncertainty remains on its performance in keratoconic eyes. While some studies reported high reliability in evaluating the corneal curvature in keratoconus^19,20^, others, based on the examination of elevation data, showed poor repeatability^21^. In our study, the repeatability of tomography data was significantly lower in keratoconic eyes than in the control group (Table 1). This finding was true when assessing the anterior and posterior surfaces and in the estimations of curvature and astigmatism (M, p = 0.64, J_0_, p = 0.01, J_45_, p = 0.53). However, while a high repeatability of an instrument’s measurements is an indication of its precision, measurements with low repeatability should be interpreted with caution. This is due to the possible misalignment between successive measurements, which may be due to unavoidable variations in eye alignment with the instrument.

Analysing the misalignment between successive measurements in our study showed that while individual misalignment parameters (x_0_, y_0_, z_0_ α, β and γ) were not statistically different in KC eyes compared with the control group, the combined misalignment (CM) parameter showed a wide gap between KC and normal corneas (P < 0.01). This difference could be due to the particular difficulty in locating the apex in keratoconic eyes, which may lead to the larger fluctuation observed between measurement in comparison to the control group. Further, the apex, relative to which all elevation measurements are made, may not coincide with the corneal geometric centre in keratoconic eyes because of the typical regional protrusion and skewed hemi-meridians associated with the disease. Besides, the visual acuity in KC patients was poorer than in normal eyes (p < 0.01). The resulting difficulty in fixation and apex detection could therefore behind the larger CM, and hence the reduced repeatability, in KC eyes seen in this study.

Further, since tomography measurements in the Pentacam system are based on the Scheimpflug image from the corneal surface, the clarity of the cornea is important to obtaining accurate measurements^22^. Anatomic changes reported in KC eyes, which include elongated epithelial cells at corneal apex^23^, alteration of regular arrangement of collagen fibrils^24^, and clear stromal spaces^25^ may influence the optical clarity of cornea and affect the measurement accuracy for corneal tomography. Similar to previous studies^26^, the repeatability of Pentacam data observed in this study decreased in eyes with corneal thinning and contour changes in eyes, both of which phenomenon are associated with KC progression. There is also a decrease in the corneal transparency secondary to alterations in the optical density of the stroma in KC which in turn causes increased scattering of light^27^.

In this study, an ICP algorithm, developed in earlier work^10^ was used to estimate misalignment between each two successive tomography measurements. Correction for the small misalignments detected resulted in significantly reduced matching errors between successive maps from 18.43 ± 21.54 μm to 6.35 ± 4.58 μm (p < 0.01) in anterior KC maps and from 29.53 ± 24.62 μm to 19.62 ± 11.79 μm (p < 0.01) in posterior KC maps. In normal controls, the errors also reduced from 5.12 ± 3.07 μm to 2.83 ± 1.12 μm (p < 0.01) in anterior maps and from 12.66 ± 5.20 μm to 11.08 ± 4.72 μm (p < 0.01) in posterior maps. Therefore, while correcting for misalignment significantly improved the repeatability of all measurements, there were residual errors which may be caused by optical distortion (possibly due to aberrations in Pentacam’s measuring lens), measurement noise, and reduced accuracy in peripheral and posterior corneal regions.

The misalignment ratio, which is intended to quantify the part of the matching error caused by misalignment, was higher in KC eyes (55.20 ± 19.99% and 27.01 ± 16.83% in anterior and posterior surfaces, respectively), compared with 38.92 ± 17.59% and 12.54 ± 11.4% in normal controls. A further trend is the lower misalignment ratio seen in posterior than anterior surfaces, which may be caused by changes in corneal transparency or corneal refractive index^27^. These changes may have influenced the image resolution of tomography and amplified the effect of misalignments on corneal repeatability (PosICP-RMS increased in KC than control groups). The irregular surface and reduced transparency of the anterior cornea may also affect posterior region data acquisition and its interpretation^28^.

All the matching results for anterior corneal surface were correlated with keratoconus grade demonstrated that the repeatability of tomography measurements on Pentacam was lower for more advanced keratoconus than for early keratoconus, which was consistent with a previous study^29^. The correlation between repeatability and the grade of keratoconus needs consideration when attempting to identify disease progression in order to make decisions for patients in relation to surgical intervention.

To our knowledge, this is the first report that evaluates the repeatability of corneal tomography measurements in keratoconic eyes and considers the effect of possible misalignment. Compared with normal eyes, KC showed higher misalignment errors, possibly causing which reduced data repeatability. The misalignment’s effect was more pronounced in estimation of astigmatism than spherical curvature. Misalignment errors also correlated with keratoconus severity.

## Methods

### Study participants

Data were analyzed for 143 eyes of 143 KC patients (108 male and 35 female, age 21.32 ± 5.51 years), and the same number of eyes of 143 gender- and age-matched, healthy subjects (108 male and 35 female, age 22.23 ± 4.32 years) who were recruited into the study at the Eye Hospital of Wenzhou Medical University. After complete clinical and imaging examinations, one independent corneal specialist (SHC) confirmed the diagnosis of keratoconus based on the criteria^26^: corneal topography showing an inferior steep spot or an asymmetric bow-tie pattern with or without skewed axes, at least one slit-lamp findings (apical thinning, Munson sign, Fleischer ring, Vogt striae and Rizutti sign). All subjects were able to fixate well at the designated target. The key exclusion criteria for both KC and healthy groups included wearing soft contact lenses within 2 weeks of involvement in study or wearing rigid contact lenses within 4 weeks, corneal astigmatism greater than 3.00 diopters (D) (except in the KC patients), corneal scarring or a prior history of surgical intervention such as corneal ring implantation, lamellar surgery or penetrating keratoplasty.

Further, the Tomographic Keratoconus Classification (TKC) system provided by the Pentacam software (OCULUS Optikgerate GmbH, Wetzlar, Germany) was used for keratoconus classification as indicated in previous studies^30,31^. The TKC offers a classification system with 5 grades: 0 (normal) to 4 (severe keratoconus). Where in some cases intermediate grades (eg, 2–3) are displayed, the lower value was recorded^30,31^. Participants in the KC group had a TKC grade between 1 and 4, while members of the healthy group had a TKC grade of 0 in addition to satisfying the same gender and similar age conditions of match with the healthy group.

Data from only one randomly-selected eye of each participant was collected, where the randomization was based on a random number sequence (dichotomic sequence, 0 and 1) that was created with Excel 2010. The study followed the tenets of the Declaration of Helsinki and was approved by the Scientific Committee of the Eye Hospital of WenZhou Medical University. Signed informed consent for online, open-access publication of images or information was obtained from all participants after explaining the procedures to them.

### Data Acquisition

All participants underwent a standard ocular examination including slit-lamp microscopy, fundus examination, manifest refraction and tomography measurement. Best corrected visual acuity (BCVA) was recorded in LogMar units, and manifest refractive error (RE) was measured with a phoroptor (Nidek RT-2100; Nidek Inc, Gamagori, Japan) in the conventional notation of sphere, negative cylinder, and cylindrical axis. The tomography data included corneal elevation maps of anterior and posterior surfaces provided by a Pentacam (OCULUS Optikgerate GmbH, Wetzlar, Germany). During data acquisition, subjects were instructed to fixate on the internal fixation lamp with room lights switched off. The device was moved back and realigned again after finishing each acquisition. Tomography measurements were taken by the same trained examiner (LFH), while the details were described in previous studies^10,32^. All methods were performed in accordance with the relevant guidelines and regulations.

### Repeatability Analysis

Iterative Closest Point (ICP) method, a feature-based registration and surface matching technique, was directly applicable to the featureful 3D shape of the corneal anterior and posterior surfaces. It was utilized to estimate and correct for misalignment between successive tomography measurements, as described in a previous study^10^. Misalignment was characterized by three translational parameters (x_0_, y_0_ and z_0_) and three rotational parameters (α, β and γ), along with the combined misalignment parameter (CM) developed to synthesize the effect of all six misalignment components^10^.

The root mean square (RMS) of the difference in elevation data pre (PreICP-RMS) and post (PosICP-RMS) tomography matching based on the ICP algorithm between two successive tomography measurements was determined^10^. Further, a misalignment ratio, calculated as (1 - PosICP-RMS/ PreICP-RMS), was used to describe the part of the error between two successive measurements that is caused by misalignment.

### Corneal keratometry calculation

In order to evaluate the effect of misalignment on the corneal tomography measurements, corneal curvature and astigmatism in the central 3 mm zone were calculated before and after correction for misalignment. According to the principal curvature method^33,34^, corneal keratometry was expressed in the form of M(x,y), the local spherical equivalent of corneal optical power, J_0_(x,y), the local cylinder at 0-degree meridian and J_45_(x,y), the local cylinder at 45-degree meridian. The distribution of corneal power vector across the aperture comprises the power vector map. A numerical integration method was then adopted to calculate M, J_0_ and J_45_, which represent the average values of M(x,y), J_0_(x,y) and J_45_(x,y), respectively, over a circular corneal aperture of 3 mm in diameter. The three parameters were intended to provide measures of spherical power and astigmatism, and enable comparisons of corneal curtvature before and after correction for misalignment.

### Statistical analysis

The comparison of tomogrphy matching results between KC and control groups were tested by the Mann-Whitney U test, while Wilcoxon test was ultilized to compare the RMS and keratometry results before and after correction for misalignment. Data analysis was conducted using statistical software SPSS 20.0 (Chicago, USA) and a P value <0.05 was considered to be statistically significant. The correlation between the keratoconus grade and the tomography matching results was determined by Spearman correlation analyses. Using software G*power for Windows (version 3.1.2, Franz Faul, Christian-Albrechts-Universität Kiel, Kiel, Germany), the sample size was calculated while an α of 0.05 and a power of 0.95 for Wilcoxon-Mann-Whitney tests. The calculations showed that a sample size of at least 110 for each group was needed.
